# Melancholia as a Form of Insanity

**Published:** 1905-04-29

**Authors:** W. Lloyd Andriezen

**Affiliations:** late Deputy Medical Superintendent, Darenth Asylum


### Hospital Clinics.

### MELANCHOLIA AS A FORM OF INSANITY.

By W. Lloyd Andriezen, M.D.Lond., late Deputy Medical Superintendent, Darenth Asylum.

The term melancholia is applied by some writers with great latitude of meaning to any condition of mental depression, with lowering of the spirits and diminished feeling of well-being, which may occur in persons sane or insane. The looseness of such terminology has created, especially in the region of psychological medicine, much confusion of thought, for the most varying and divergent kinds of mental diseases, whose fundamental clinical features, etiology, course, pathology, and prognosis differ essentially, have in turn been made to serve the office of a *corpus vile* and been dubbed melancholia because the patient exhibited temporary or brief phases and periods of "melancholic depression" in the course of his illness. The condition of melancholia comprises, it is true, a number of characteristic symptoms—viz., a depression of spirits and of the general feeling of well-being, a sense of feebleness and of fatigue with little capacity for exertion, a mood of anxious apprehension, and a brooding over fancied impending evil which pervades the mental atmosphere like a gloom even though a fair or full degree of mental lucidity is preserved. Physically there are associated disturbances of the digestive and assimilative processes of health, dryness and coldness of the skin and extremities, loss of appetite often with heartburn, insomnia, and a dull headache with more or less tenderness of the scalp, a hard pulse with high radial tension, constipation, and ischuria. The melancholiac loses interest in duties and pleasures alike, forsakes his habitual occupations, is introspective and absorbed in the painful thoughts and anxious fears of a vague character which besiege his mind. It is the combination of these elements and their persistence and development to such an extent as to endanger the mental life of the individual that constitute melancholia. There may not be the least external cause, or but very slight cause, for the profound apprehensive mental depression of the melancholiac, and if any cause be traced it will be found to be out of all proportion, insignificant in relation to the grave reaction present mentally and bodily in the patient. Even when the cause has passed away the melancholia will continue to persist, increase in intensity, to reach its climax, and then only to subside after tedious and slow remissions. In all but the mildest cases of simple melancholia more or less potent delusions occur of a painful, or hypochondriacal, or persecutory type, and, as a consequence, suicidal desires and longings, frequently leading to suicidal attempts, occur. In the more acute forms, almost without exception, the patient has previously been reduced to a state of profound physical debility as a result of recent illness, long-continued defective digestion, improper food, want, and defective hygienic surroundings, or anæmia. The melancholiac has been, as a rule, a person of good physique and of more than average sensibility and intelligence; usually a "finely-developed" brain is the one most liable to develop melancholia in its classical form.

Melancholia is more frequent among women than men, as their habits and life encourage conditions favourable to its development. The disease should be considered as one of adult life and of middle or later life, the vast majority of cases occurring between the ages of 35 and 60 years. It is rare in adolescents, and almost unknown in children. In women the disease appears a little earlier than in men, seeming to bear a relation to the mental involution of the climacterium, while in males the onset tends to be later. A defective or psychopathic heredity is found in a little over one-half of the patients. Apart from hereditary predisposition the most frequent causes are business worry and anxiety, great financial losses, grief from deaths or other domestic calamities, and such debilitating processes as starvation, over-fatigue, puerperal and lactational exhaustion, and post-febrile anæmia and exhaustions. Gastro-intestinal disorder with auto-intoxication from the bowel is now recognised as playing an important contributory part in the onset and course of melancholia.

The prodromal stages of melancholia vary in different subjects. In some persons the depression of spirits, the mental anxiety, and the constitutional weakness never pass the limits of sanity and transient malaise. I have personally known of two cases of "simple depression of spirits" occurring in persons two or three times a year for a lifetime, each attack lasting four or five days and completely passing away. Perfect mental lucidity is preserved in both cases: one is a man of 55, in whom I observed this regularly for the past 12 years and who states that he had been so affected all his life; the second case, a man of 50, is a somewhat similar instance. Both are quite sane, and I am certain, from long and intimate knowledge of these two cases, that neither of them will ever lapse into insanity. Dr. Lange, of Copenhagen, has stated in a memoir published in 1886 that a condition of "simple depression of spirits may occur in some persons periodically or at regular intervals, and may continue throughout life without passing into actual melancholic or other forms of insanity," and my observation and experience quite confirm his view. The famous writer, Thomas Carlyle, was probably a classical example of it. Putting aside this variety of periodic depression of sane subjects, we pass to the prodromes of the true melancholia of insanity. The result is gradual, insidious, and usually marked by anorexia, constipation, and gastro-intestinal disorder. A persistent tendency to worry in regard to health, business, or family affairs, an aversion for work and a disregard of personal tidiness may have existed for some time. The patient's mood becomes seclusive and gloomy, but no suspicion is excited in the minds of friends or relatives of the oncoming danger until an attempt at suicide or other dangerous act suddenly calls attention to the condition of mental alienation which had insidiously established itself. In some cases the onset may be of rapid occurrence, following upon serious shock or mental stress in a predisposed subject, as in the case of a neurotic girl who, after seduction and desertion, passes into a condition of insomnia, physical prostration, and grave melancholia. A woman at or near the period of her menopause, when profound involutional changes are developing in the nervous and reproductive system, is liable to develop melancholia with delusions of marital infidelity more readily than at other times. As melancholia tends to become established, nutritive defects of the body become more evident. The skin becomes of a dry muddy tint, and feels harsh and dry to the touch. Perspiration is very slight or absent. A slight degree of jaundice may exist. The mouth and lips are dry, the tongue furred and flabby, the bowels are constipated, and the breath tends to be offensive. Gastric digestion is feeble, appetite fails, and weight is lost. The urine is small in quantity, shows a trace of albumen, and is loaded with phosphates. The pulse is slower than normal, the arteries are contracted, and the radial tension is high. Sleep is profoundly disturbed, a condition of distressing insomnia being prevalent, combined with frightful dreams and nightmare. Headache, tenderness of the scalp and nuchal spine, feelings of paræsthesiæ and fleeting neuralgias in various parts of the body become prominent symptoms. In women the menstrual process is arrested or the normal flow is decreased. The mental symptoms include slowness in thought and ideation, vague anxiety and apprehensions, disinclination for work or initiative of any kind, undue liability to fatigue, and mental confusion as the result of enforced mental exertion, while the mental atmosphere is one of misery, gloom, and despair. Mental lucidity persists for some time, and the sense of orientation in regard to time and place is at first preserved. Mental lucidity, however, disappears as delusions set in. The delusions are at first slight and fleeting, but tend to persist, and become well-marked after a while. Such delusions as predominate are of a very painful character; they are characteristic only of the severer forms of melancholia. The concentration of the mind upon the *ego*, and the inactivity of the kinæsthetic life, tend to foster moods favourable to delusions. Among the commoner forms of delusion are those of being poisoned, of being damned and in hell, of having committed the sin against the Holy Ghost or other unpardonable offence, of having committed murder, of being in a state of ruin and disgrace, of being a burden and shame to those near and dear, and generally of being wicked and deserving of punishment, and meriting all suffering. The mental attitude and tone are humble, lowly, self-abasing, a contrast to the grandiose, persecutory delusions and defiant attitude of the paranoiac. In more advanced cases delusions may develop to the extent that they involve negations of the whole or part of the *ego* or body, the patient saying, "This is not I," the "bodily organs are another's," etc.—(Cobard's *delire de negation*). The prognosis then is hopeless.

As regards general prognosis, simple melancholia may end either in recovery within a few weeks or may pass into a graver stage. In the latter case the patient becomes more depressed and self-absorbed, refuses food and medical treatment, and develops delusions. The stage of vague fear and apprehension has passed into one of confirmed despair and misery, and delusions, which were at first occasional and fleeting, now become powerful and persistent. Hardly any two patients present exactly the same delusions and the latter are hard to classify. They may, however, be grouped into two main classes—namely, hypochondriacal (somatic or visceral) delusions, and delusions of pain, fear, and disgrace, among which delusions of sins against religion are prominent. Delusions of voices uttering insults and imprecations, and of people in the street mocking and abusing the patient are frequently entertained. Illusions and hallucinations of taste and smell are excited by disordered secretions of the mouth and nose, and from such illusions the mental transition is easy to delusions of poison being administered in the food or in the form of "vapours" by enemies. In hypochondriacal melancholia the delusions refer to the stomach, bowels, lungs, heart, genitalia, limbs, hair and nails, skin, or other parts. Delusions of being infected by loathsome diseases, such as syphilis or cancer, of having gangrene of an organ or a limb, or of being infested with parasites and worms, comprise the list of hypochondriacal complaints. Patients of this class are pictures of abject misery, wholly absorbed in their complaints and loud in expatiating on them, and suicidal attempts often are made, not necessarily to obtain relief from suffering, but to gain attention, sympathy, and notoriety. In agitated melancholia the patients manifest incessant movement, such as tearing of the hair, wringing of the hands, sobbing and weeping, striking of the forehead and breast with the hands, and repeated interjections of woe. Ideas of eternal damnation have a peculiar fascination for such patients, and as long as the state of agitation exists the patient remains thin and anæmic. When recovery or a lapse into dementia is about to occur patients commence to grow fat.

As regards differential diagnosis it is important to distinguish the involutional melancholia of adult and middle life from the temporary melancholic and quixotic exhibitions, shallow and histrionic, of dementia præcox, and to avoid confusion between the graver degrees of melancholia with delusions and the melancholic form of general paralysis of the insane. The profound mental deterioration and special physical signs of the latter should render diagnosis easy. The delusions of paranoia are characterised by their special stamp—*e.g.* the mental lucidity attached to the period of their evolution, their evolution into logical "systems" of delusions, and the proud and defiant megalomania of paranoia, in contrast with the abject and self-abasing attitude of the melancholiac.

